# Life Habits of Healthcare Professionals during the Third Wave of COVID-19: A Cross-Sectional Study in a Spanish Hospital

**DOI:** 10.3390/ijerph20054126

**Published:** 2023-02-25

**Authors:** Enedina Quiroga-Sánchez, Natalia Calvo-Ayuso, Cristina Liébana-Presa, Bibiana Trevissón-Redondo, Pilar Marqués-Sánchez, Natalia Arias-Ramos

**Affiliations:** 1 SALBIS Research Group, Department of Nursing and Physiotherapy, Campus of Ponferrada, University of León, 24400 Ponferrada, Spain; 2 Department of Nursing and Physiotherapy, Campus of Ponferrada, University of León, 24400 Ponferrada, Spain

**Keywords:** health personnel, sleep quality, sleep hygiene, health behaviour, feeding behaviour, COVID-19

## Abstract

(1) Background: To describe sleep quality, eating behaviour and alcohol, tobacco and illicit drug use among healthcare staff in a Spanish public hospital. (2) Methods: Cross-sectional descriptive study examining sleep quality (Pittsburg Sleep Quality Index), eating behaviour (Three Factor Eating Questionnaire (R18)), tobacco and drug use (ESTUDES questionnaire) and alcohol use (Cut down, Annoyed, Guilty, Eye-opener). (3) Results: 178 people, of whom 87.1% (155) were women, with an average age of 41.59 ± 10.9 years. A total of 59.6% of the healthcare workers had sleep problems, to a greater or lesser degree. The average daily consumption was 10.56 ± 6.74 cigarettes. The most commonly used drugs included cannabis, occasionally used by 88.37%, cocaine (4.75%), ecstasy (4.65%) and amphetamines (2.33%). A total of 22.73% of participants had increased their drug use, and 22.73% had increased their consumption during the pandemic, with beer and wine accounting for 87.2% of drinks consumed during this period. (4) Conclusions: In addition to the psychological and emotional impact already demonstrated, the COVID-19 crisis has repercussions on sleep quality, eating behaviour and alcohol, tobacco and drug consumption. Psychological disturbances have repercussions on physical and functional aspects of healthcare workers. It is feasible that these alterations are due to stress, and it is necessary to act through treatment and prevention as well as promote healthy habits.

## 1. Introduction

In the healthcare field, the different stress-generating situations have multiple repercussions, not only on business operations but also on the health of the patient and the workers involved. With this in mind, reviews of the literature describe how exhaustion derived from work stress affects the worker’s commitment to the organization, their productivity, patient safety and satisfaction and the quality of care [1,2]. Similarly, it can also modify the health behaviours of the worker and push them away, in certain situations, from the recommended guidelines [3].

Along these same lines, sleep is one of the factors that most contribute to physical and psychological well-being. Nursing professionals are usually, within the healthcare community, the most affected by sleep disorders [4]. In fact, those who suffer from this type of alteration have a high risk of developing performance decreases and making errors when administering medication [5]. Likewise, sleep disorders are related to the presence of multiple health problems [6]. An example of this is the consequences derived from the alteration of the circadian rhythm that occurs in workers who have night shifts or irregular shifts. Going even further, it has been possible to verify the relationship between this type of alteration and the presence of diabetes mellitus [7], cardiovascular diseases, metabolic syndrome or cancer [8].

Another consequence of suffering from sleep disorders is the limitation that those affected have in managing stress [9]. In this sense, although it has been seen how nurses (specifically) have a series of coping resources that can be classified as healthy (for example, socialization), they can also activate other resources that are not beneficial for their health, such as alcohol and tobacco use, social avoidance or anger displacement [10]. Regarding the consumption of alcohol, tobacco and illicit drugs, Baldisseri et al. (2007) [11] estimated that approximately 10% to 15% of healthcare professionals will abuse drugs or alcohol at some point in their professional life. In fact, they demonstrated a higher rate of abuse of benzodiazepines and opiates. Blake et al. (2011) [12] found that, of a sample of 325 nurses, two-thirds exceeded the recommended maximum daily intake of alcohol, and nearly one-fifth were smokers. A review of the literature carried out by Nilan et al. (2019) [13] reported a prevalence of tobacco use in nursing between 21% and 25%, varying according to the socioeconomic level of the country.

Similarly, shift work, as an example of a stress-causing agent, negatively influences dietary habits and the weight of workers, increasing the prevalence of obesity. A higher frequency of food intake and/or consumption of poor-quality food has also been noted among shift workers [14,15,16]. The emotions that are generated in complex situations are capable of modifying eating behaviour, marking, for example, certain preferences for some foods or even modifying caloric intake [4].

In Spain, the SARS CoV 2 COVID-19 pandemic hit hard. By 30 April 2021, 78,216 people had died, and more than 80,000 healthcare workers had been infected [17]. This situation has meant that healthcare workers in our country have faced numerous work stressors, such as long working hours and/or work overload, among others. As a consequence, wave after wave, levels of anxiety and depression have progressively increased in nursing staff [18], and so have multiple sleep disorders in the healthcare community [19], with a significant impact on both physical and mental health [20,21,22,23,24]. Given healthy lifestyles among healthcare professionals may be compromised by the multiple consequences of the current pandemic, it is necessary to explore what impact the third wave of COVID-19 has had on the health of our healthcare professionals. Thus, the aim of this present study was to describe the sleep quality, eating behaviour and alcohol, tobacco and drug consumption of healthcare workers in a Spanish public hospital. In doing so, we aim to highlight the importance of health-related habits, as well as the need to promote strategies to improve these habits and, consequently, the well-being of these workers.

## 2. Materials and Methods

### 2.1. Design

An intervention-free cross-sectional descriptive study was proposed, carried out in the months of February to March 2021 through an online, anonymous, and completely voluntary questionnaire and developed through the Google Forms^®^ application. With the aim of reaching the largest possible number of healthcare workers in the shortest possible time, the dissemination was carried out through an instant messaging platform (WhatsApp).

The STROBE checklist guidelines for observational research have been followed.

### 2.2. Participants and Selection Criteria

All healthcare personnel of legal age, who had a working relationship with the health centre, were considered to participate. As an inclusion criterion, the healthcare personnel had to present the informed consent document covered and signed. The final sample was made up of 178 subjects.

### 2.3. Measurements and Instruments

Several sociodemographic variables were considered (age, sex, marital status, work service, seniority, type of contract, professional category and type of cohabitation), and through the Pittsburgh Sleep Quality Index (PSQI), sleep quality was evaluated. This questionnaire validated in Spanish [25] contains a total of 24 items that are grouped into 7 dimensions, which provide information on the different factors that affect sleep quality. Thus, subjective sleep quality refers to the subject’s assessment from 0 (very bad) to 3 (very good). Similarly, sleep latency measures how long the subject thinks it takes them to fall asleep, while sleep duration reports the actual number of hours a person sleeps at night. The efficiency of habitual sleep results from the percentage relationship between the time the subject believes they are asleep and the time they have been lying down. On the other hand, sleep disturbances inquire about the frequency with which alterations are noticed. Finally, it also contemplates the use of sleep medication and daytime dysfunction, understood as the impact of sleep on the development of daytime activities. The graphic representation of the scores obtained in each of the components allows us to clearly see where the problems related to sleep lie. The sum of the scores of the 7 dimensions gives a score varying from 0 to 21 points (the higher the score, the worse quality of sleep). Setting a cut-off point of 5 points for its interpretation, a difference is made between good sleep quality (scores below 5) and poor sleep quality (higher scores) [25].

Eating behaviour was measured using the Three Factor Eating Questionnaire (R18) (TFEQ-R18). This validated tool [26] consists of 18 items with a Likert-type response model with 4 possible options from 1 (rarely) to 4 (always). Thus, it evaluates three dimensions of eating behaviour: uncontrolled intake (tendency to eat more than usual due to loss of control when eating with a subjective sensation of hunger); emotional eating (the inability to resist emotional cues or eat in response to negative emotions); and cognitive restriction (the conscious restriction of eating aimed at controlling body weight and/or promoting weight loss). The three domains are converted to a scale from 0 to 100 (de Lauzon et al., 2004) according to the following equation [(raw score−lowest possible raw score)/possible raw score range) × 100]. Thus, higher scores indicate a higher probability of the domain to which they refer. This test presents appropriate reliability coefficients for the three subscales (ranging from 75 to 85) that are also indicated in a nursing population (85 to 90) [27].

Related to tobacco use (daily use, in the last 30 days, in the last year and starting age) and use of drugs that are illegal in Spain (use, type of drug and use in the last 12 months), the ESTUDES-validated questionnaire (Survey on Drug Use in Secondary Education in Spain) belonging to the National Drug Plan was used. This validated tool aims to collect information on drug use and other addictions in order to design and evaluate policies aimed at the prevention of this type of substance and the problems derived from it, mainly focused on the family and/or school environment. A total of 10 items referring to the consumption of said substances (6 items on tobacco and 4 on illicit drugs) were selected for this study.

Finally, data on alcohol consumption were collected using the CAGE (Cut down, Annoyed, Guilty, Eye-opener) validated questionnaire [28,29] to detect cases of alcohol dependence or abuse. Developed by Ewing (1984) [28] and validated by Mayfield et al. (1974) [30], it is characterized by its brevity, simplicity and ease of application. It comprises a total of 4 questions, which can be administered in the context of a clinical interview or in isolation. Each affirmative answer adds 1 point so that the existence of problems is evidenced when 2 or more questions are answered affirmatively. It has a sensitivity between 65–100% and a specificity of around 88–100% [31,32].

### 2.4. Data Analysis

A descriptive analysis of the variables described was carried out using the SPSS statistical package in version 26.0. The description of the variables analysed was carried out using measures of central tendency and dispersion, in the case of quantitative variables, mean and standard deviation (SD). Qualitative variables were expressed as absolute frequencies and percentages. The normality of the variables was tested using the Kolmogorov–Smirnov test with Lilliefors modification.

### 2.5. Ethical Considerations

This study was approved by the Research Ethics Committee of the Health Areas of León and Bierzo (registration number: 20205) and the Ethics Committee of the University of León (ETICA-ULE-044-2020). Likewise, it was designed in such a way as to respect the ethical principles for global medical research that are reflected in the Declaration of Helsinki and its subsequent modifications. Prior to participation in the study, it was specified through informed consent documents that participation was completely voluntary, making it clear that the exploitation of the registered data would be carried out completely anonymously and confidentially for research purposes.

## 3. Results

A total of 178 people participated, out of which 87.1% (155) were women, with a mean age of 41.59 ± 10.9 years, with 22 being the minimum and 68 the maximum. Table 1 shows the descriptive data of the sample.

**Figure 1:**
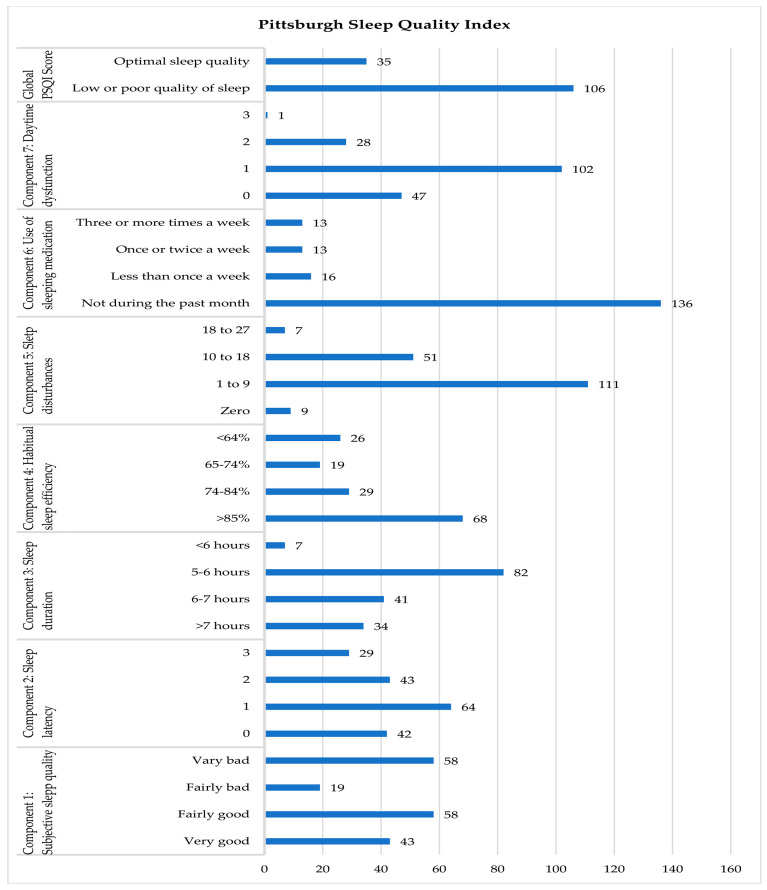
Descriptive statistics of the Pittsburgh Sleep Quality Index (PSQI).

Figure 1 shows the graphic representation of each of the PSQI components. Thus, 59.6% (106) of the healthcare workers presented sleep problems of greater or lesser importance. A total of 19.7% (35) of participants showed optimal levels of sleep quality, measured by the PSQI. In Table 2, we can see the descriptive results of each of the PSQI components.

**Figure 2:**
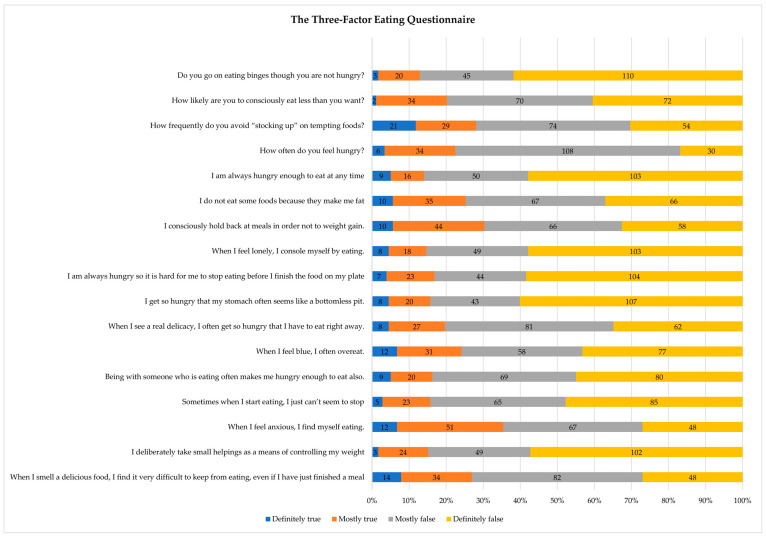
Descriptive statistics of the Three Factor Eating Questionnaire (R18) (TFEQ-R18).

Descriptive data on eating behaviour according to the Three Factor Eating Questionnaire (R18) (TFEQ-R18) are described in Table 2 and Figure 2. The results of the dimensions of “uncontrolled intake” and “emotional eating” are highlighted as they were exceptionally high (74.45 ± 20.50 and 70.6 ± 25.78 out of 100, respectively).

Regarding the consumption of toxic substances, tobacco, alcohol and illicit drugs were considered for this study. About tobacco use, according to ESTUDES, the average age of onset is 16.23 ± 2.67 years, with 9 being the minimum age of onset and 29 being the maximum. Meanwhile, the average daily consumption was 10.56 ± 6.74 cigarettes (with a minimum of 1 and a maximum of 30 cigarettes per day with a frequency of consumption mainly daily in 25.28% (45) of the participants but also sporadic, weekly in 5.06% (9) and even less in 3.37% (6).The most used illicit drugs were cannabis, used at some point by 88.37% (38), followed by cocaine in 4.75% (2) of the participants, ecstasy (4.65% (2) and, finally, amphetamines used at some point by 2.33% of the respondents). On the other hand, regarding alcohol, 22.73% (25) had increased its consumption during the pandemic, with beer and wine representing 87.2% of the drinks consumed in this period. Cocktails (3.67%), liquors (0.92%) and other types of alcoholic substances (8.26%) were also consumed. In Table 3, we can see more extensively the data referring to the consumption of tobacco, drugs and alcohol.

All respondents consumed alcohol. In terms of consumption pattern analysed using the CAGE questionnaire, we noted that 94.94% (169) were social drinkers. Risk consumption was also observed by 2.25% (4). Likewise, the existence of harmful consumption was noted in 1.69% (3) of the respondents and alcohol dependence in 1.12% (2) of the participants.

## 4. Discussion

The literature has shown that the work dynamics of healthcare personnel generate high levels of stress and exhaustion, with important implications for health, both physical and mental [20,21,22,23,24], a situation that has been aggravated due to the COVID-19 pandemic [17]. This research has addressed aspects that interfere not only with the biopsychosocial development of the individual but also with the work quality of healthcare personnel during COVID-19. Thus, sleep quality, general and emotional eating behaviour and the consumption of alcohol, tobacco and illicit drugs have been studied.

Our study confirmed that the characteristics of healthcare professionals are similar to those of other studies carried out in care units [33,34]. The sociodemographic data show that 87.1% of the sample were women with a mean age of 41.6 years, with the female gender being represented in more than 80% of health-related jobs. Healthcare workers are mainly women with an average working age superior to 40 years [35]. In addition, 68.5% turned out to be nursing professionals, making it the largest group among the employees surveyed. Indeed, nurses rank as the largest healthcare workforce, representing more than 50% of the total healthcare workforce globally [35,36]. In spite of this, these figures are far from reaching the ideal levels to offer quality care since a greater number of nurses are needed to guarantee optimal health levels [37]. Along the same lines, the employment profile of the hired personnel has been described. The results of this study show that only 36% of healthcare personnel have a tenure, which they obtained by passing an examination for Public Service, while 64% have a temporary employment contract. However, it is true that the comparison with other countries can be somewhat complex as labour contracts vary depending on the health scenarios. Thus, a Brazilian study prior to the pandemic informs us that, indeed, most healthcare professionals were hired as service providers (temporary staff) [38]. In this line, the results of this work highlight the Spanish problem related to the shortage of healthcare personnel. Observed by public administrations and in an attempt to deal with this pandemic, staff from the different existing job placement offices and newly graduated professionals (mostly novices) were hired to help with the health emergency situation. The enormous spreading speed of COVID-19, added to the high number of infections among healthcare personnel, has forced these administrations to increase the supply of temporary jobs [39,40,41].

Regarding the quality of sleep, the results of this study showed that around 60% of healthcare personnel have some sleep disorder, while around 20% reflect optimal levels. This data is similar to those found in other comparable works, in which healthcare personnel are presented as a group with poor sleep quality, not only before the pandemic but also after [42,43]. Although the proportions obtained in this study are high, other investigations that evaluated the sleep quality among healthcare personnel during COVID-19 show even higher percentages. For example, in one of the most important studies carried out during the pandemic period, it was found that 75% of healthcare workers had poor sleep quality [44]. Similarly, in Saudi Arabia, using the same questionnaire, a prevalence of sleep deprivation of 83% was found among healthcare personnel [45]. If it were not for the pandemic period, this fact could be justified by the alteration of circadian rhythms derived from rotating shifts (usual shifts in care units), which in turn causes less stable sleep rhythms [46]. In the current context, the authors think it is important to point out that the high prevalence of sleep disorders found in this study, conducted one year after the start of the pandemic, may be related to concerns related to the contagious nature of COVID-19 and the work dynamics established in this period. On the other hand, when comparing the overall PSQI score of our work (7.92 ± 4.18) with other studies, we can see how our figures are lower than those obtained in other investigations. For example, in China, in a study carried out on 180 healthcare workers who worked during COVID-19, in addition to presenting a higher PSQI score (8.6 ± 4.6), they also showed high levels of stress and anxiety had a negative impact on sleep quality [47].

Furthermore, regarding emotional eating, this study observed that the sample presented a high level of emotional eating (the inability to resist emotional signals or eat in response to negative emotions) (70.6 ± 25.8). Likewise, the data obtained regarding uncontrolled intake (74.4 ± 20.5) (tendency to eat more than usual due to loss of control when eating with a subjective feeling of hunger) were high. In other studies, these data were obtained in response to negative or uncomfortable emotional states [48]. Regarding cognitive restriction (conscious restriction of eating aimed at controlling body weight and/or promoting weight loss), although the results are better compared to the other dimensions of the questionnaire (66.3 ± 16.3), they are also high. Job changes, job demands and uncertainty reflect how healthcare personnel “compensate with food” the negative emotions experienced in stressful situations [21].

In relation to tobacco consumption, our results show how one out of three healthcare professionals increased their consumption during this third wave. The published literature has already indicated that cigarette consumption increased in Spanish healthcare professionals during COVID-19 [49]. This may be motivated by the increase in cigarette consumption in the face of environmental stressors of various kinds, such as conflicts, disasters in the presence of depressive symptoms or post-traumatic stress disorders [50,51].

As for alcohol consumption, in this work, it is noted that one out of every four healthcare workers has increased consumption. Although such consumption has been associated with social life for decades, the pandemic has shown that its absence has not led to a reduction in alcohol consumption. In the healthcare environment, alcohol abuse or dependence may be associated with having been working as a healthcare personnel during the pandemic period and may again be related to situations that potentially generate post-traumatic stress disorders [50,51,52].

Here, we present the limitations and future lines of research. This study shows a series of limitations to highlight. The first is the sample size and having carried out this study in a single hospital. Furthermore, the difficulty in answering the Pittsburgh questionnaire can be considered another limitation. Indeed, our results show 37 people did not complete this instrument.

In future lines of study, we propose to expand this research with the inclusion of not only other variables of interest that have not been contemplated yet but also professionals from other centres from different parts of Spain and even Europe. It would also be interesting to include healthcare professionals from hospital centres as well as those dedicated to community health in the study sample.

## 5. Conclusions

The literature reports that the COVID-19 crisis has affected all types of healthcare workers, generating a significant emotional impact. The feeling of fear, anxiety or uncertainty, as well as the care overload or the pressure to which healthcare workers have been subjected during the pandemic, has considerably increased the consequences on their physical and psychological health. This study provides data on the quality of sleep and diet and the consumption of alcohol and tobacco of healthcare professionals in times of the pandemic. It is reflected in our results that the alterations already demonstrated in psychological health transcend other fields of the physical sphere of the individual and that they are basic for their functioning not only as workers but also as people. Since it is thought that stress levels could be the cause of such affectation, it would be interesting to implement strategies not only dedicated to the prevention and treatment of psychological consequences but also address fundamental aspects that favour healthy habits.

## Figures and Tables

**Table 1:** Descriptive statistics of the sample.

Sociodemographic Variables		Number of Participants n = 178	Percentage (100%)
**Sex**	Female	155	(87.1%)
	Male	23	(12.9%)
**Marital Status**	Married	88	(49.4%)
	Divorced	9	(5.1%)
	In a couple	43	(24.2%)
	Separated	4	(2.2%)
	Widowed	1	(0.6%)
**Work service**	COVID-19 service	33	(18.5%)
	Non-COVID-19 service	97	(54.5%)
	Occasionally COVID-19	48	(27.0%)
**Years of work**	Less than 5 years old	61	(34.3%)
	From 5 to 10 years	24	(13.5%)
	More than 10 years	93	(52.2%)
**Employment contract**	Statutory/State employee	64	(36.0%)
	Temporary	57	(32.0%)
	Interim	57	(32.0%)
**Professional category**	Orderly	7	(3.9%)
	Nurse	122	(68.5%)
	Medical Doctor	10	(5.6%)
	Auxiliary Nursing Care Technician	39	(21.9%)
**Type of cohabitation**	Alone	17	(9.6%)
	With family	92	(51.7%)
	Couple/Friends	69	(38.8%)

Note: n, absolute number; %, percentage.

**Table 2:** Descriptive statistics, mean, standard deviation, minimum and maximum of Three Factor Eating Questionnaire (R18) (TFEQ-R18) and Pittsburgh Sleep Quality Index (PSQI).

		Number of Participants n = 178 (M ± SD)	Percentage Min–Max
**TFEQ-R-18**	Uncontrolled intake	74.45 ± 20.50	7.41–100
	Emotional eating	70.6 ± 25.78	0–100
	Cognitive restraint	66.26± 16.29	27.78–94.44
**PSQI**	Component 1: Subjective sleep quality	1.52 ± 1.18	0–3
	Component 2: Sleep latency	1.33 ± 1.01	0–3
	Component 3: Sleep duration	1.36 ± 0.86	0–3
	Component 4: Habitual sleep efficiency	1.33 ± 1.26	0–3
	Component 5: Sleep disturbances	1.31 ± 0.63	0–3
	Component 6: Use of sleeping medication	0.46 ± 0.92	0–3
	Component 7: Daytime dysfunction	0.90 ± 0.66	0–3
	Global PSQI Score	7.92 ± 4.18	1–19

Note: n: absolute value; M: mean; SD: standard deviation; Min: minimum; Max: maximum.

**Table 3:** Frequencies (n) and percentages (%) of responses to the ESTUDES and CAGE questionnaires referring to the use of tobacco, illegal drugs and alcohol.

Tobacco and Illegal Drugs (ESTUDES Questionnaire)	Yes % (n)	No % (n)
**1. Have you ever smoked cigarettes in your life?**	32.6% (136)	76.4% (42)
**5. In the last 12 months, have you smoked more than usual?**	24.2% (43)	52.2% (93)
**6. Do you consider that tobacco consumption has increased during the pandemic?**	30.9% (55)	45.5% (81)
**7. Have you ever used any illegal drug?**	24.2% (43)	75.8% (135)
**9. In the last 12 months, have you used any illegal drug?**	5% (5)	95% (38)
**10. Has illegal drug use increased during the pandemic?**	4.65% (2)	95.35% (41)
**Alcohol (CAGE Questionnaire)**		
**Has alcohol consumption increased during the pandemic?**	22.7% (25)	77.3% (85)
**1. Have you ever felt that you should drink less?**	11.8% (21)	88.2% (157)
**2. Has it bothered you when people criticise you for your drinking?**	3.9% (7)	96.1% (171)
**3. Have you ever felt bad or guilty about your drinking?**	3.9% (7)	96.1% (171)
**4. Have you ever needed to drink in the morning to calm your nerves or relieve discomfort from drinking the night before?**	2.2 (4)	97.8% (174)

Note: n, absolute number; %, percentage.

## Data Availability

The data presented in this study are available upon request from the authors. Data are not publicly available due to privacy and ethical restrictions.
